# Sangerbox 2: Enhanced functionalities and update for a comprehensive clinical bioinformatics data analysis platform

**DOI:** 10.1002/imt2.238

**Published:** 2024-09-02

**Authors:** Di Chen, Lixia Xu, Huiwu Xing, Weitao Shen, Ziguang Song, Hongjiang Li, Xuqiang Zhu, Xueyuan Li, Lixin Wu, Henan Jiao, Shuang Li, Jing Yan, Yuting He, Dongming Yan

**Affiliations:** ^1^ Department of Neurosurgery The First Affiliated Hospital of Zhengzhou University, Zhengzhou University Henan China; ^2^ Department of Infectious Diseases The First Affiliated Hospital of Zhengzhou University, Zhengzhou University Zhengzhou China; ^3^ Department of Pediatric Surgery The First Affiliated Hospital of Zhengzhou University Zhengzhou China; ^4^ Bioinformatics R&D Department Hangzhou Mugu Technology Co., Ltd Hangzhou China; ^5^ The First Department of Cardiology The First Affiliated Hospital of Harbin Medical University Harbin China; ^6^ Department of MRI The First Affiliated Hospital of Zhengzhou University Zhengzhou China; ^7^ Department of Hepatobiliary and Pancreatic Surgery The First Affiliated Hospital of Zhengzhou University, Zhengzhou University Zhengzhou China

**Keywords:** batch analysis, bioinformatics, data processing, web server

## Abstract

In recent years, development in high‐throughput sequencing technologies has experienced an increasing application of statistics, pattern recognition, and machine learning in bioinformatics analyses. SangeBox platform to meet different scientific demands. The new version of Sangs is a widely used tool among many researchers, which encourages us to continuously improve the plerBox 2 (http://vip.sangerbox.com) and extends and optimizes the functions of interactive graphics and analysis of clinical bioinformatics data. We introduced novel analytical tools such as random forests and support vector machines, as well as corresponding plotting functions. At the same time, we also optimized the performance of the platform and fixed known problems to allow users to perform data analyses more quickly and efficiently. SangerBox 2 improved the speed of analysis, reduced resource required for computer performance, and provided more analysis methods, greatly promoting the research efficiency.

## Highlights

- SangerBox 2.0 has expanded its features by adding new machine learning tools, including random forest and support vector machine (SVM), with improved plotting capabilities.
- The platform's performance and visualization tools have been significantly optimized, including the introduction of interactive adjustments for heatmaps.
- SangerBox 2.0 stands out in bioinformatics analysis due to its enhanced multifunctionality, user‐friendliness, and superior performance compared to other platforms.

## Graphical abstract

This abstract showcases the key enhancements of SangerBox 2, focusing on versatility, performance optimization, rich visualization tools, user‐friendliness, and enhanced interactivity. The platform integrates advanced machine learning tools, improves computational efficiency, and introduces new visualization options like wordclouds and Manhattan plots. Interactive features allow real‐time heatmap adjustments, greatly enhancing the user experience. SangerBox 2 supports both public and personal data, utilizing cloud storage and computing, making it a versatile tool for bioinformatics research.

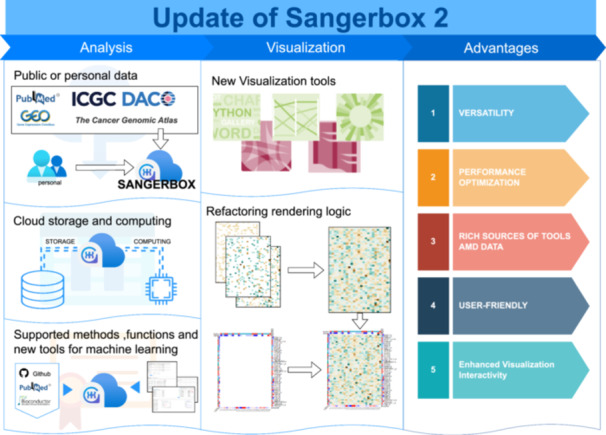

## INTRODUCTION

The exponential growth of data generated during biomedical research covers a wide range of dated from genomes, transcriptomes, and proteomes and has become an invaluable resource for researchers to gain deeper insights into biological processes and disease mechanisms; yet, efficiently processing and analyzing these complex data is a huge challenge for most researchers. To meet everyone's needs in all aspects, a variety of data analysis and visualization platforms have been developed, such as ImageGP [1], Majorbio Cloud [2], and our SangerBox [3], a comprehensive and user‐friendly clinical bioinformatics analysis tool to facilitate researchers to more efficiently analyze their scientific data.

**Figure 1:**
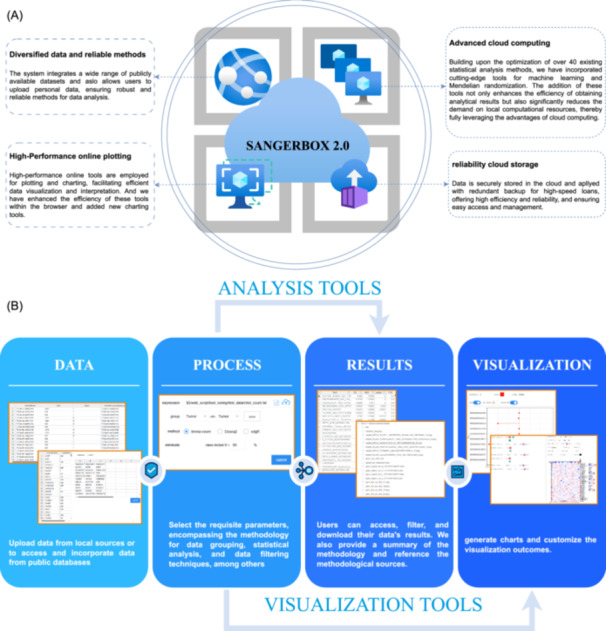
Overview of SangerBox 2. (A) SangerBox's user‐friendly accessibility is underpinned by a robust foundation of diverse data integration, validated analytical methods, expeditious and rational cloud computing services, state‐of‐the‐art online visualization techniques, and trustworthy cloud storage solutions. (B) The standard workflow encompasses a sequence of steps. Initially, users are prompted to upload or select data, with the flexibility to opt for publicly available data sets or to submit their proprietary data. Subsequently, they are invited to choose methodologies, set parameters, and preprocess the data. Upon completion of the analysis facilitated by the analytical tools, users are then able to peruse, select, and download the outcomes pertinent to their research needs. Ultimately, with plotting tools or the acquired results, users are endowed with a suite of conventional graphical representations that can be interactively tailored to their specifications.

Since the introduction of SangerBox, the user‐friendliness and multifunctionalities have become the two most outstanding characteristics of the platform. Therefore, it has been widely employed in a variety of biological and medical research projects, gaining increasing reputation in the relevant research filed. To better accommodate constantly progressing and changing research directions, technologies, and application scenarios, the platform is undergoing a series of adjustments and optimizations (Figure 1A). Moreover, its novel analytical functions are added to meet different demands of researchers.

Present SangerBox 2 is the latest version of the platform. Compared with the initial version, we made several updates to address the current research hotspots and challenges in bioinformatics and clinical medicine. The new version fixed the known problems in previous versions and optimized the mechanism of the graphics software, improving the overall performance of the platform. Moreover, we added new interactive graphics tools and some commonly used graphics and tables. Researchers are now empowered to perform data analysis with enhanced simplicity and efficiency (Figure 1B), facilitated by our streamlined approach that significantly reduces the complexity traditionally associated with research studies. As for the current research hotspots, machine learning such as random forest and their corresponding graphics tools were also supplemented.

The development of SangerBox 2 was motivated by the increasing need for a more versatile and user‐friendly bioinformatics platform capable of handling the complexity of modern omics data [4]. Traditional platforms, while powerful, often demand extensive computational resources and specialized knowledge, creating a barrier for many researchers. SangerBox 2 addresses these challenges by integrating advanced machine learning tools and optimized visualization capabilities, enabling users to perform sophisticated analyses with greater ease and efficiency. Additionally, its cloud‐based architecture supports scalable data processing, which is crucial for managing the large data sets prevalent in current research. Compared to other platforms like Bioconductor, SangerBox 2 offers a more intuitive interface and faster processing times, making it an ideal choice for both novice and seasoned bioinformaticians.

The concept of SangerBox 2 is to provide researchers with a powerful, efficient, reliable, and easy‐to‐use platform that allows them to focus on their research without paying more attention to the details of data processing. It also offers a variety of efficient visualization features to smoothly process the big data in bioinformatics on devices of average performance (Figure 1A).

## RESULTS

### Case study

**Figure 2:**
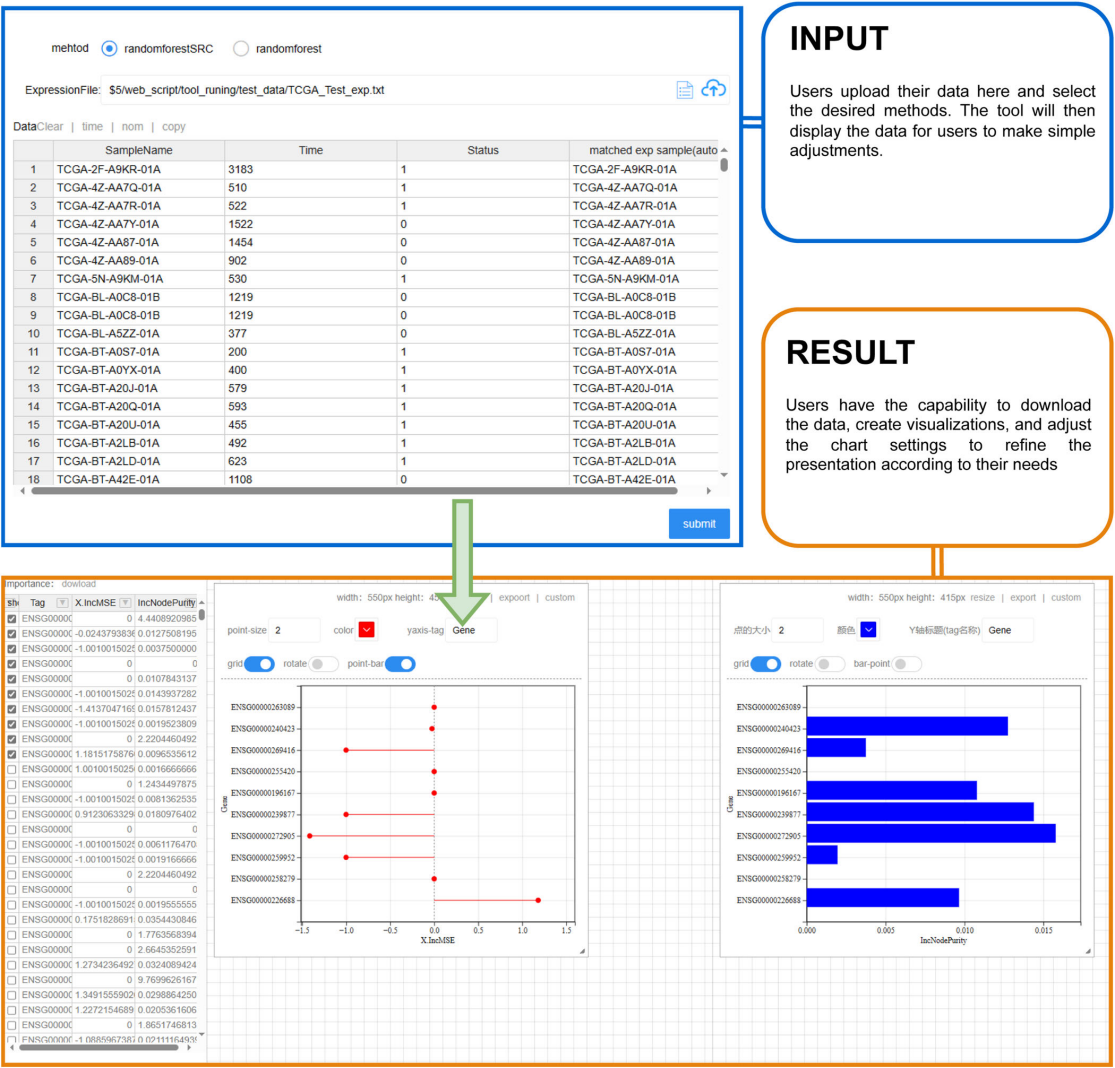
Random forest tool analysis case.

In our exemplified random forest tool (Figure 2), users initiate the process by selecting a preferred analytical approach, either “randomforest” or “randomforestsrc.” Subsequent to their selection, users are prompted to upload their matrix data, with a default assumption of transcriptomic expression profiles, yet with the flexibility to substitute alternative matrix types, such as omics or clinical data sets. Following this, the platform facilitates the replication of the dependent variable data for the samples. As demonstrated, we have opted for the “randomforestSRC” method and entered the survival data, whereupon our system conducts a thorough check for data formatting discrepancies, including sample name alignment. Post submission, users await the analytical outcomes. Once the results are ready, a results matrix is presented on the left panel, granting users the capability to download and selectively filter the data. Furthermore, users are endowed with the functionality to generate a variety of graphical representations, offering customization options such as color schemes, graphical styles, label rotations, and renaming capabilities to tailor the visualization to their specific requirements.

### Performance benchmarks

We have built upon SVG and D3.js [5] to optimize the generation logic and code, enabling high‐performance vector graphics rendering in the browser. The performance evaluation is primarily based on the following metrics: (i) Rendering speed: The speed at which the drawing tool renders vector graphics in the browser, ensuring quick responses even with large data sets. (ii) Resource utilization: The central processing unit and memory usage of the drawing tool during operation, ensuring efficient performance on standard computers. (iii) Interactivity: The smoothness of user interactions when adjusting parameters and visualizing data, ensuring a seamless and efficient user experience. (iv) Output quality: The quality and accuracy of exported graphic files in various formats, meeting the needs for academic publication and presentation. By optimizing algorithms and implementing efficient code, we ensure that these drawing tools can run smoothly on general computers without the need for high‐performance computing devices. Based on these performance metrics, the Sangerbox 2 drawing tool provides an excellent user experience and high‐quality output in practical use.

## DISCUSSION

### Comparative advantages

Compared with other bioinformatics analysis platforms, SangerBox 2 demonstrates significant advantages in terms of multifunctionalities, user‐friendliness, and performance optimization. SangerBox 2 integrates advanced analytical tools such as random forest and support vector machines (SVMs), as well as highly efficient visualization tools that enable researchers to explore data more comprehensively. Although Galaxy provides a broad range of tools, it lacks the real‐time interactivity and advanced machine learning integration that SangerBox 2 offers. Bioconductor, though comprehensive, often presents a steep learning curve due to its reliance on the R programming language. In contrast, SangerBox 2 combines the accessibility of a graphical user interface with powerful computational tools, making it more approachable for users with varying levels of expertise. Additionally, the platform's ongoing updates ensure it remains at the forefront of bioinformatics innovation. As the field continues to evolve, SangerBox 2 is well positioned to incorporate emerging technologies, such as single‐cell RNA sequencing and multi‐omics data integration, further solidifying its role as a critical resource for researchers globally.

### Evolution of software and platform tools

We believe that the upgrade of software and platform follows a process of upward spiral. Each update is based on user feedback and technological advances, with the goal of gradually adding new features and optimizing existing ones. In the future, we will continue to expand the functions of our platform to improve better user experience and data analysis capabilities.

### Open API initiative

Although our original intention was to facilitate general nontechnical users, we also welcome other developers to contribute to the program. In the future, we plan to provide open API interface to allow users and developers to customize and extend the functionality of the platform according to their own needs. Open APIs will facilitate the integration of the platform with other tools and databases, offering a more comprehensive and flexible solution.

### Need for user feedback

Though SangerBox 2 demonstrates an outstanding performance in a number of ways, we are aware of the need to constantly improve the functions of our platform. For us, user feedback is such an important source of the continuous improvement. We sincerely invite users to provide valuable comments and suggestions during their use of SangerBox 2 to help us identify potential problems and offer directions for further improvement.

## CONCLUSION

SangerBox 2.0 introduced advanced analysis methods such as random forest and SVMs, as well as graphics tools, significantly enhancing the functions and user experience of the platform. The multifunctionalities and user‐friendliness have allowed SangerBox 2.0 platform to become an indispensable analytical tool in bioinformatics research. Looking ahead, the platform's impact on bioinformatics is poised to grow as more researchers adopt the platform for their data analysis needs. Its user‐friendly interface, coupled with robust analytical tools, makes it accessible to a broad spectrum of scientists, from those in academia to industry professionals. The platform's capability to handle both public and proprietary data sets, supported by cloud storage and computing, ensures its applicability in diverse research contexts. As user feedback continues to inform its development, future updates are likely to introduce even more advanced features, such as AI‐driven data analysis and enhanced collaboration tools, further increasing its utility in the rapidly advancing fields of genomics and beyond.

Our user feedback indicated that Sangerbox 2.0 has achieved a high performance in terms of data analysis accuracy, processing speed, and resource utilization. The platform provides a rich resource of toolkits and efficient procedures, greatly promoting the efficiency and quality of scientific research. In the future, SangerBox 2.0 will be continuously expanded and optimized to accommodate the evolving research needs in bioinformatics studies.

## MATERIALS AND METHODS

### Analysis function

In addition to retaining its traditional functionalities, the platform has integrated several machine learning methods in response to current hot research topics. This enhancement enables researchers to more swiftly engage in cutting‐edge studies, thereby reducing the learning curve associated with these advanced techniques.

Random forests are an ensemble learning technique that harnesses the power of multiple decision trees to perform classification or regression tasks. Each constituent tree is trained on a randomly selected subset of the data set, with the selection of split points also being stochastic. In the realm of bioinformatics, random forests have found extensive applications in the analysis of gene expression data [6], protein prediction, disease classification [7], and the identification of biomarkers [8]. For instance, in the context of gene expression analysis, random forests can assist in pinpointing genes associated with specific diseases, thereby providing crucial insights for disease mechanism research and the development of novel therapeutics. We have developed a unified analytical framework by integrating the “randomForest” and “randomForestSRC” R packages, which is designed to accommodate the heterogeneous nature of user data sets. This comprehensive approach ensures that our methodology is both flexible and robust, capable of handling a wide array of data attributes and enhancing the scope of its applicability in various research scenarios.

SVMs are a powerful supervised learning technique that excels in both classification and regression tasks. SVMs work by identifying a hyperplane that maximizes the separation margin between different classes, allowing for effective discrimination of samples even in high‐dimensional spaces. In the field of bioinformatics, SVMs have been extensively used for tasks such as gene expression data classification, protein structure prediction, disease diagnosis, and biomarker discovery. For example, in protein structure prediction, SVMs can be instrumental in identifying protein features linked to specific functions, offering crucial insights into the role of these proteins in disease mechanisms. We have developed an integrated analytical framework that leverages R packages like “e1071” and “kernlab” to handle the diverse nature of user data sets. This comprehensive approach ensures that our methodology is robust and adaptable, making it suitable for a wide range of complex bioinformatics research applications.

This integration not only broadens the scope of research that can be conducted on the platform but also enhances the accessibility of complex genetic diseases analyses, making high‐level research more approachable for a wider range of investigators.

### Visualization function

**Figure 3:**
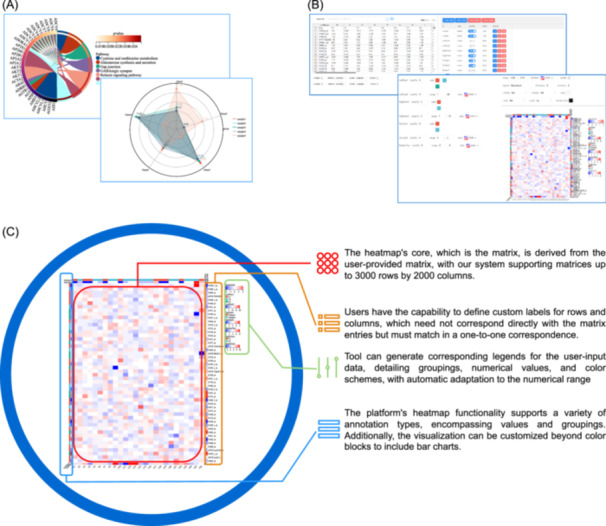
Platform's newly update plotting tool. (A) Results from the platform's newly added general plotting tool. (B) The brand new complex heatmap tool. (C) Interactive adjustment functionality within the complex heatmap tool.

We have introduced new visualization tools, including wordclouds, funnel charts, radar plot, and chordal plot as shown in Figure 3A, while simultaneously optimizing our complex heatmap drawing tools, allowing users to create customized heatmaps as shown in Figure 3B.

Our heatmap tool offers straightforward clustering methods, enabling users to customize row and column labels shown in the Figure 3C, including various types of annotation information, and perform group analysis, with automatic generation of legends. Additionally, the tool allows for interactive adjustments, where users can modify the sorting and styles within the plot through mouse clicks. This user‐friendly feature enhances the flexibility and interactivity of data visualization, providing a more engaging and personalized experience for data analysis. In our heatmap tool, we have integrated the advanced practices from existing solutions such as R packages pheatmap, superheat, ggplot2, and ComplexHeatmap [9], and retaining robust features such as extensive annotation capabilities, data segmentation, and sophisticated grouping and statistical analyses. We have further augmented these with superior interactivity and visualization performance. Notably, our tool introduces automated legends and facilitates user‐friendly adjustments to the heatmap through intuitive “click,” all within the convenience of a browser‐based interface.

Additionally, we have revised some of the underlying rendering logic for existing visualization tools. For instance, previously, charts with a large number of elements, such as volcano plots and scatter plots, could cause performance issues on devices without high‐performance graphics processors.

## AUTHOR CONTRIBUTIONS

**Di Chen**: Conceptualization; methodology; validation; funding acquisition; project administration; writing—review and editing. **Lixia Xu**: Methodology; conceptualization; visualization; validation. **Huiwu Xing**: Methodology; validation; visualization; software; data curation. **Weitao Shen**: Methodology; software; data curation; visualization; writing—original draft; writing—review and editing. **Ziguang Song**: Methodology; software; data curation; visualization. **Hongjiang Li**: Validation; visualization; data curation; investigation. **Xuqiang Zhu**: Validation; visualization; data curation; investigation. **Xueyuan Li**: Visualization; validation; data curation; investigation. **Lixin Wu**: Data curation; validation; visualization. **Henan Jiao**: Data curation; visualization; validation; formal analysis. **Shuang Li**: Software; conceptualization; data curation; resources. **Jing Yan**: Funding acquisition; conceptualization; methodology; supervision. **Yuting He**: Conceptualization; methodology; supervision. **Dongming Yan**: Conceptualization; methodology; software; project administration; validation; supervision.

## CONFLICT OF INTEREST STATEMENT

The authors declare no conflict of interest.

## ETHICS STATEMENT

Only human and animal study must have this section.

## Data Availability

Sangerbox 2 is freely available for all academic and noncommercial use. To ensure stability and quality of service, we have set reasonable limits on disk space, computing resources, bandwidth, and crawler access, based on user numbers and peak traffic periods. Access the platform at http://vip.sangerbox.com for data analysis. We encourage researchers to use Sangerbox 2 for bioinformatics studies and to provide feedback and suggestions, helping us to continually improve and optimize the platform. Supporting Information (figures, tables, scripts, graphical abstract, slides, videos, Chinese translated version, and update materials) may be found in the online DOI or iMeta Science http://www.imeta.science/. The data that support the findings of this study are available from the corresponding author upon reasonable request.
